# Supplementary figures and images for: The opportunistic intracellular bacterial pathogen Rhodococcus equi elicits type I interferon by engaging cytosolic DNA sensing in macrophages
Source: PLoS Pathog. 2021 Sep 2;17(9):e1009888. doi: 10.1371/journal.ppat.1009888 (PMC8443056; doi:10.1371/journal.ppat.1009888)

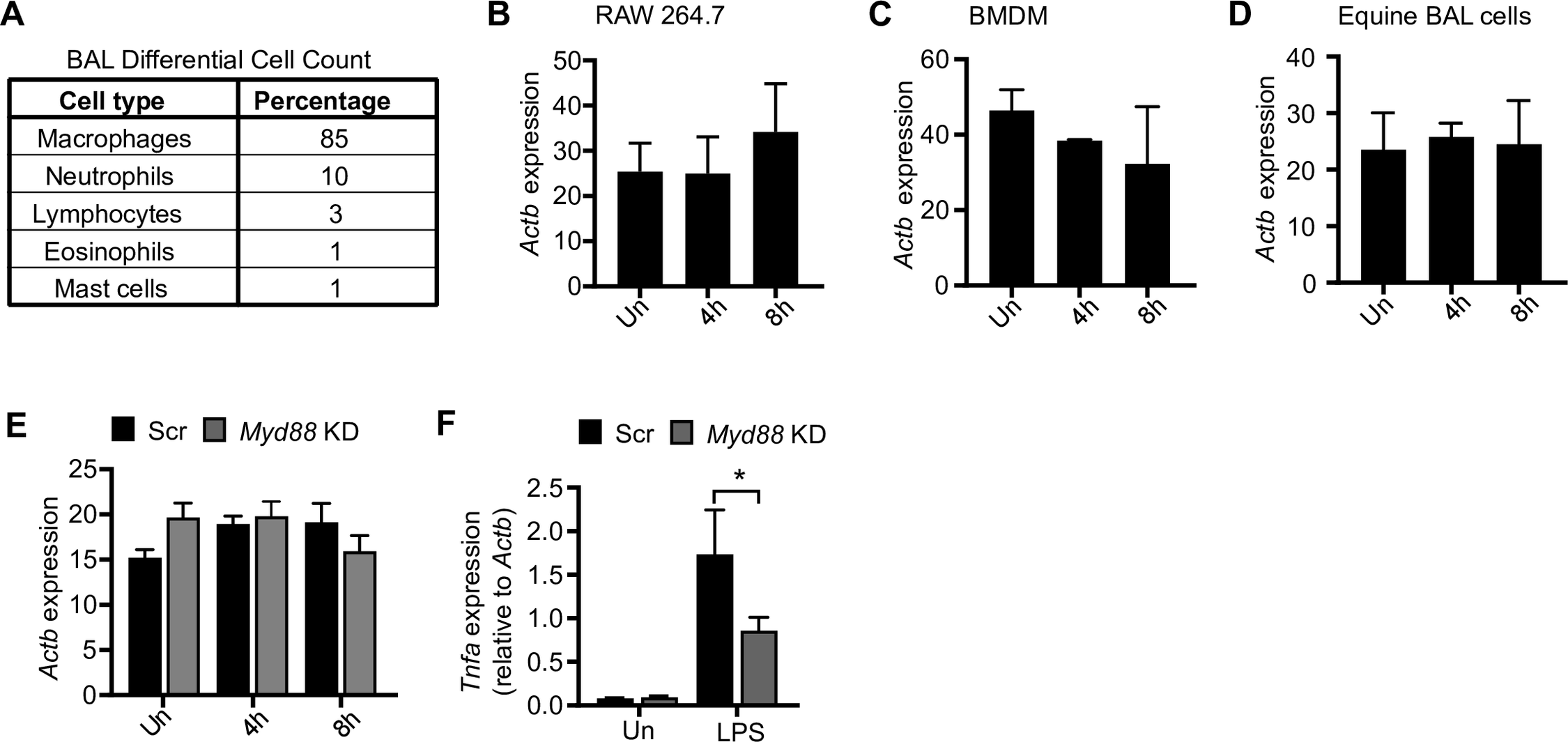

Supplement: S1 Fig — (A) Table of equine bronchoalveolar lavage (BAL) cell composition based on a 300-cell count differential. (B) RT-qPCR of Actb in murine BMDMs from experiment in Figs 1 and 2. (C) As in (B) but in RAW 264.7 cells. (D) As in (B) but in equine BAL cells. (E) As in (B) but Myd88 KD RAW 264.7 cells. (F) RT-qPCR of Tnfa in Myd88 KD RAW 264.7 cells stimulated or not with LPS for 4 hours. All RT-qPCRs are representative of at least 2 independent experiments and are the mean of 3 replicates ± SD, n = 3. (TIF) [file ppat.1009888.s001.tif]

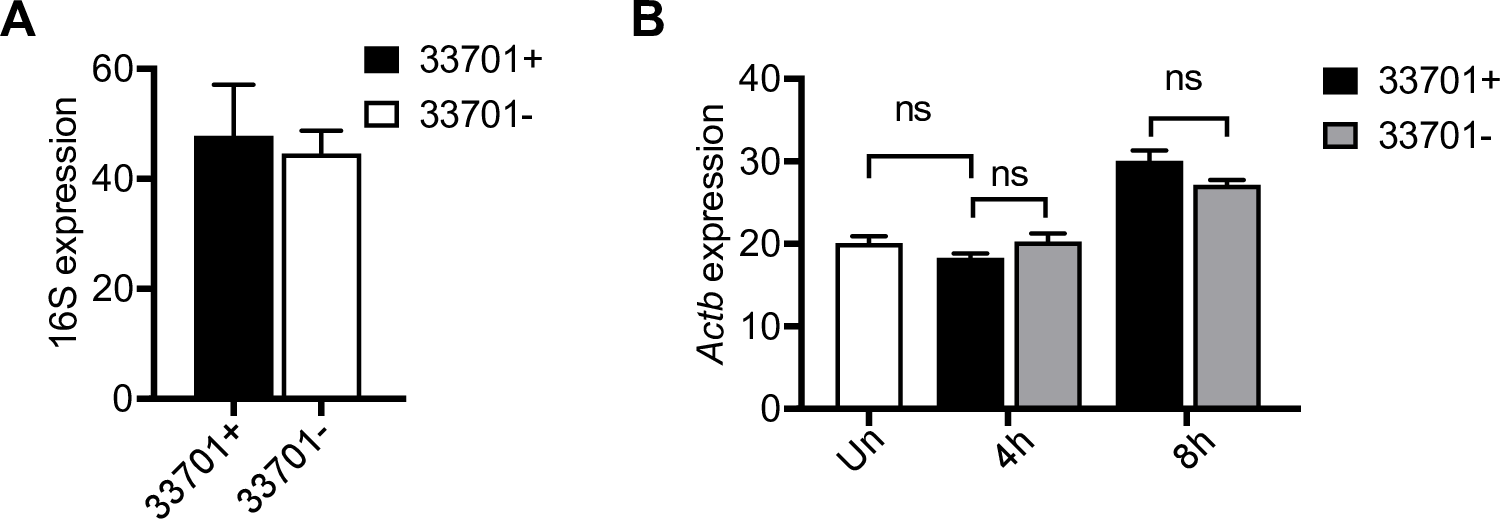

Supplement: S2 Fig — (A) RT-qPCR of 16S in R. equi 33701+ and 33701- used as a housekeeping gene for Fig 3C. (B) RT-qPCR of Actb (housekeeping gene) in RAW 264.7 experiments in Fig 3. All RT-qPCRs are representative of at least 2 independent experiments and are the mean of 3 replicates. Error bars are ± SD, n = 3. Statistical significance was determined using Students’ t-test. *p < 0.05, n.s. = not significant. (TIF) [file ppat.1009888.s002.tif]

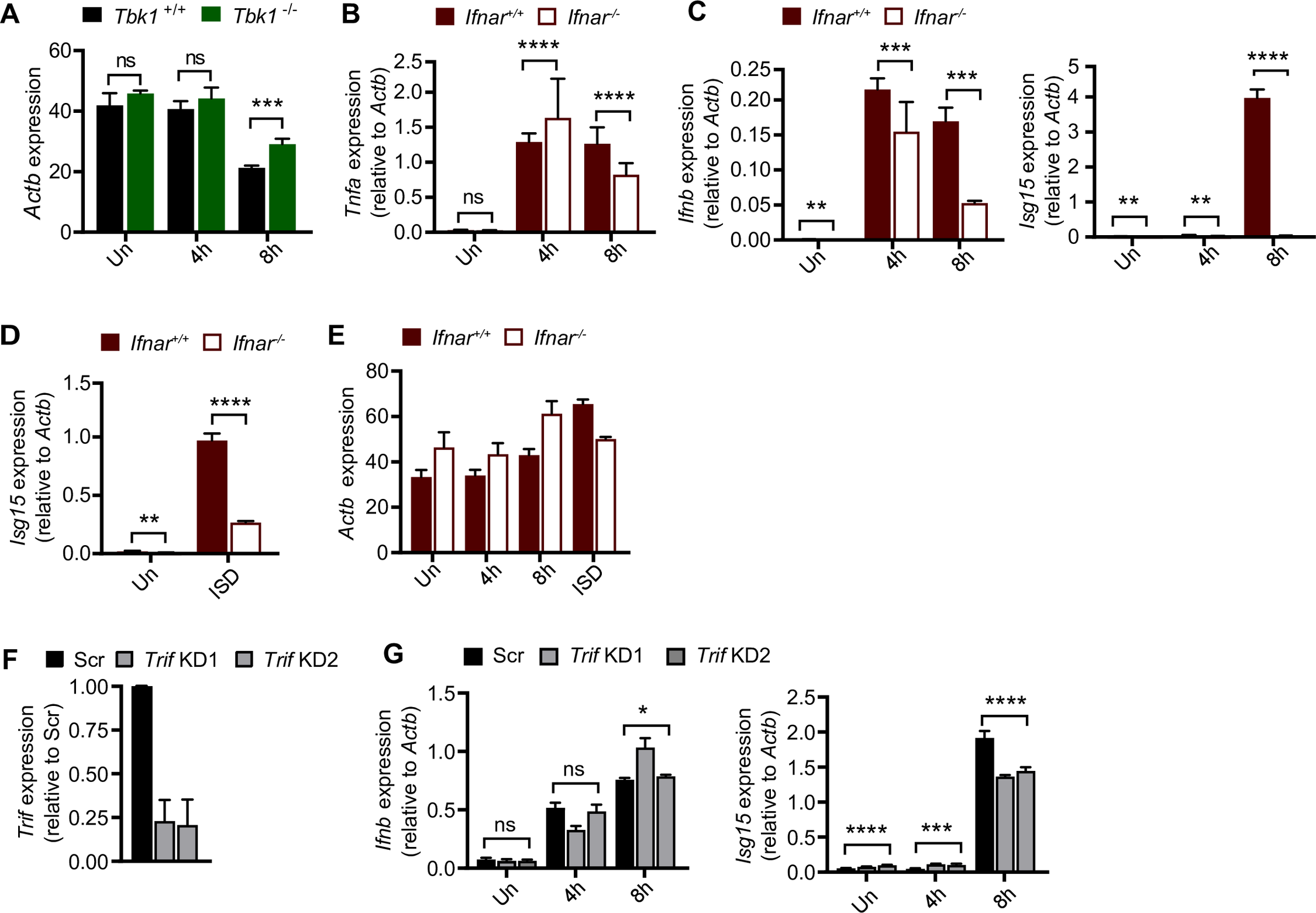

Supplement: S3 Fig — (A) RT-qPCR of Actb in R. equi-infected TBK1 BMDMs. (B) As in (A) but Tnfa in IFNAR BMDMs. (C) As in (B) but Ifnb and Isg15. (D) RT-qPCR of Isg15 in IFNAR BMDMs treated with ISD for 4h. (E) As in (B) but Actb. (F) RT-qPCR of Trif in RAW 264.7 KD cells relative to Scr control. (G) RT-qPCR of Ifnb and Isg15 in Trif KD and Scr RAW 264.7 macrophages. RT-qPCRs are representative of at least 2 independent experiments and are the mean of 3 replicates. Error bars are ± SD, n = 3. Statistical significance was determined using Students’ t-test. *p < 0.05, n.s. = not significant. (TIF) [file ppat.1009888.s003.tif]

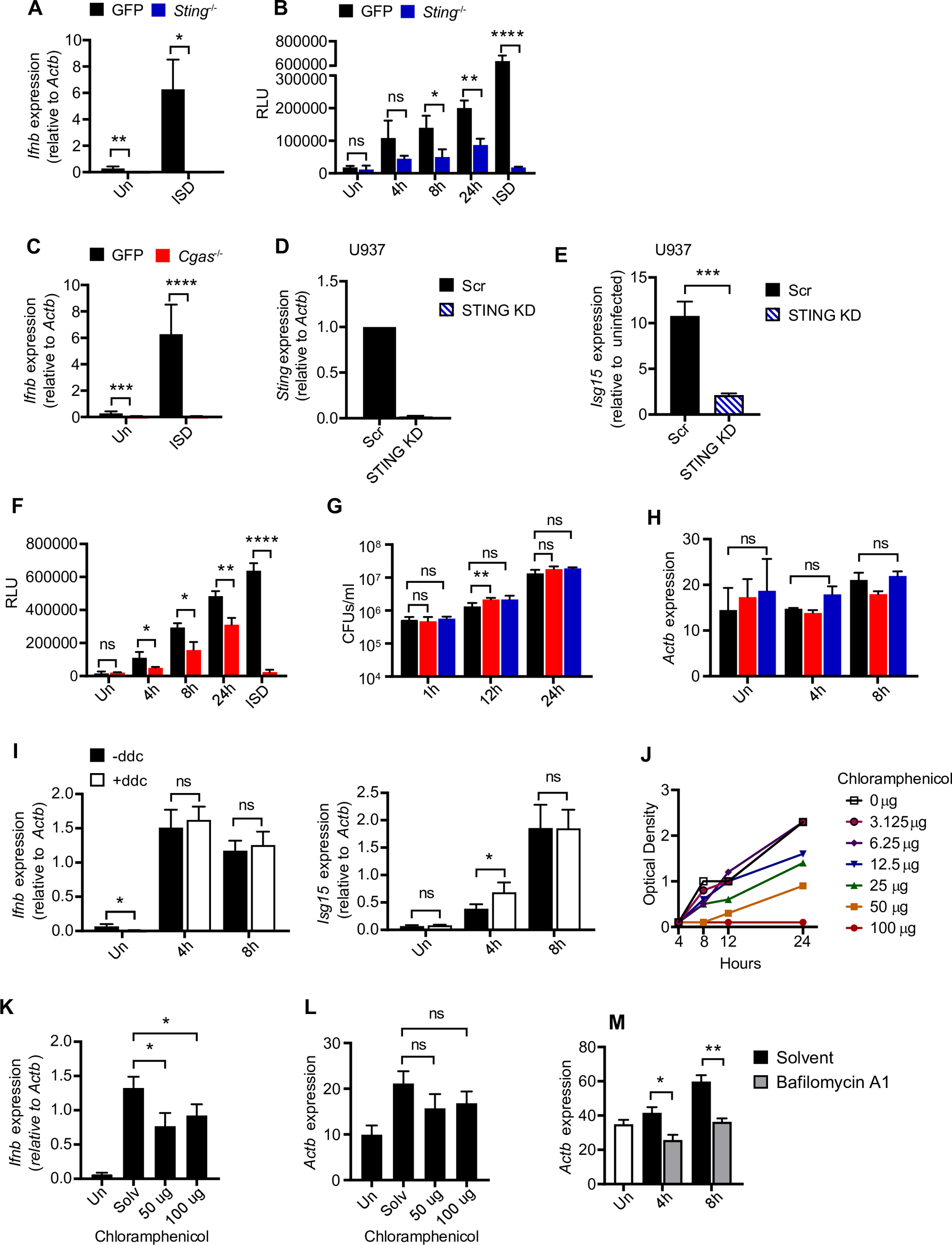

Supplement: S4 Fig — (A) RT-qPCR of Ifnb in GFP gRNA control and STING KO RAW 264.7 macrophages treated or not with ISD for 4h. (B) ISRE reporter assay measuring relative luminescence units as a readout for type I IFN protein levels secreted into supernatants from GFP gRNA control or STING KO RAW 264.7 macrophages infected or not with R. equi for the indicated times or treated with ISD for 4h. (C) As in (A) but in GFP gRNA control and cGAS KO RAW 264.7 macrophages. (D) RT-qPCR of Sting in U937 cells relative to Scr control. (E) RT-qPCR of Isg15 in STING U937 KD cells infected with R. equi for 8h. (F) As in (B) but in GFP gRNA control and cGAS KO RAW 264.7 macrophages. (G) CFUs of GFP gRNA control, cGAS KO and STING KO RAW 264.7 macrophages infected with R. equi at the indicated times. (H) RT-qPCR of Actb in GFP gRNA control, cGAS and STING KO RAW 264.7 cells at the indicated times post R. equi infection. (I) RT-qPCR of Ifnb and Isg15 in R. equi-infected RAW 264.7 cells depleted of mtDNA with 10 μM ddC for 4 days. (J) OD600 of R. equi cultures treated with the indicated concentration of chloramphenicol. (K) RT-qPCR of Ifnb in RAW 264.7 cells 8h post infection with R. equi treated with the indicated concentration of chloramphenicol. (L) As in (K) but Actb. (M) As in (L) but RAW 264.7 cells treated with 100nM bafilomycin A1. (M) RT-qPCRs are representative of at least 2 independent experiments and are the mean of 3 replicates. Error bars are ± SD, n = 3. Statistical significance was determined using Students’ t-test. *p < 0.05, n.s. = not significant. (TIF) [file ppat.1009888.s004.tif]

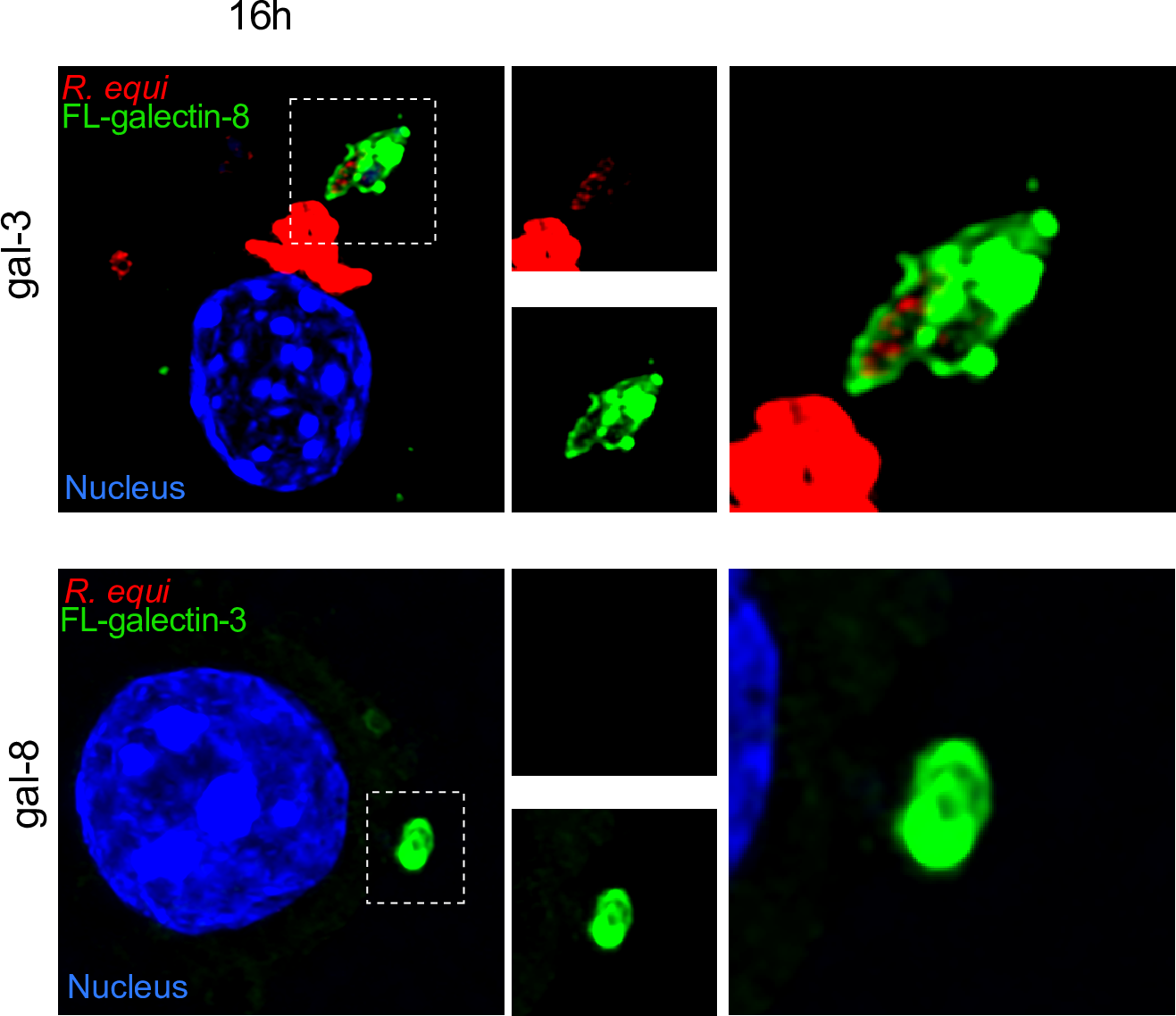

Supplement: S5 Fig — (A) Immunofluorescence (IF) of RAW 264.7 cells stably expressing 3XFLAG (FL)-tagged galectin-3 or-8 infected with GFP expressing R. equi 103+ (MOI-5) for 16h. (TIF) [file ppat.1009888.s005.tif]
