# Supplementary material for: The opportunistic intracellular bacterial pathogen Rhodococcus equi elicits type I interferon by engaging cytosolic DNA sensing in macrophages
Source: PLoS Pathog. 2021 Sep 2;17(9):e1009888. doi: 10.1371/journal.ppat.1009888 (PMC8443056; doi:10.1371/journal.ppat.1009888)
Supplement: Attachment — Submitted filename: R. equi Response to Reviewers.docx [file ppat.1009888.s007.docx]

Response to Reviewers

**Comments from Reviewers:**

Reviewer #1: The study by Vail et al, entitled “The opportunistic intracellular bacterial pathogen *Rhodococcus equi* elicits type I interferons by engaging cytosolic DNA sensing in macrophages” demonstrates that *R. equi* activates cytosolic DNA sensing in macrophages and elicits type I IFN responses in vitro and in vivo and it its potential impact in *R. equi* *in vivo*. This is an interesting study showing where similar results are observed in *R. equi* akin to what has been also described for M. tuberculosis. Said this, this study is important but there are some clarifications that authors must address and/or justify better.

Reviewer #2: The opportunistic intracellular bacterial pathogen *Rhodococcus equi* elicits type I interferons by engaging cytosolic DNA sensing in macrophages
Review on the work from Vail et al.,
In their study the authors investigated the mean by which the bacterial pathogen *Rhodococcus equi* is able to be detected and trigger a Type-I interferon pathway in its target cells, namely macrophages. The authors found that *Rhodococcus equi*-induced type I IFN-dependent ISG production requires cGAS/STING/TBK1 cytosolic pathway which suggests that this bacterium needs a direct or indirect access to the host cell cytosol. To this regard, the authors found that phagosomal permeabilization markers (Galectin 3, 8 and 9) were significantly recruited around several intracellular *Rhodococcus equi*, which suggests that through a yet to be determined mechanisms a small proportion of *Rhodococcus equi* has access to the host cell cytosol. In order to determine which virulence mechanisms/effectors might promote such response, the authors tested the well characterized virulence effector (VapA) encoded on the bacterium virulence plasmid but did not find it required at modulating cGAS/STING/TBK1 activation path. Finally mouse and foal infections showed a robust ISG and IFN production pathway.
Overall, the study is well designed, executed and the findings that *Rhodococcus equi* also triggers a CGAS/STING path in a similar way to some Mycobacteria, the pathogen *F.tularensis* spp novicida etc… add a novel stone in the understanding of how microbial species interact to the intracellular environment.
Two important questions remain unfortunately not addressed in this study:
Is the activation of the cGAS/STING/IFN path important for the cell autonomous immunity of macrophages or for the general fight of the immune system against *Rhodococcus equi*?
Using mice or macrophages lacking cGAS or IFNAR or STING might help the authors to answer such question.
Another standing question from the study is if the putative ESX-encoded secretion system from Rhodococcus equi is able to trigger cGAS activation and phagosomal escape/destabilization? Is there a way the authors could try such approach (genetic against some putative compnents of this ESX?), as it would bring important scientific information regarding the general virulence components important for driving such process

We’d like to start off by thanking the reviewers for their supportive, constructive comments. We have done our absolute best to address the major critiques, both by making changes to the manuscript and through additional experiments.

**Part II – Major Issues: Key Experiments Required for Acceptance**

Reviewer #1: 1/ For the RNA-seq study, the use of a mouse tumor cell line is understandable but removes the importance of using mouse primary alveolar macrophages to count for the lung environmental effects during infection, or perform an ex vivo analysis using isolated AMs after infection in mice. It seems that there is a disconnection between the RNA-seq results and the rest of the study. The discussion is not bringing together Fig 1 data with the rest. This study could easily start in Fig 2 defining the Th1/Th2/Th17 responses to *R. equi* during infection in vitro (or in vivo).

We agree with many of the valid points made here. First, to address concern re: the disconnect between the RNA-seq results and the rest of the study, we rearranged our figures. Figure 1 is focused on demonstrating that *R. equi* induces pro-inflammatory cytokines with similar kinetics in three difference cell types: primary bone marrow derived macrophages (as a primary macrophage model) (Fig. 1A), equine bronchoalveolar macrophages (BAL) (as a cell type more representative of the major *R. equi* host) (Fig. 1B), and RAW 264.7 macrophage cell line (Fig. 1C-D) (as a genetic model moving forward). ***The data from equine BAL cells is completely new since our original submission***. We then use these data, which show that these three different types of macrophages induce proinflammatory gene expression to similar levels/with similar kinetics, to motivate our RNA-seq experiments using the RAW 264.7 macrophage cell line in Figure 2. In addition, we added references from our work and others that have shown that RAW 264.7 cells behave similarly to primary macrophages with regard to TLR and cytosolic sensing events during Mtb, *Salmonella*, and *Listeria* infections (lines 129-131). We feel the RNA-seq data is important because it is what alerted us to the type I IFN response being activated during *R. equi* infection. We hope that the new text (lines 125-134, 158-159, 417-418), new equine BAL data (Fig. 1B), and new organization address your concerns.

As a last point, we tried several times to isolate enough alveolar macrophages to do a proper *R. equi* infection and were unsuccessful. However, since there is no major indication that RAW 264.7 cells are not an appropriate model for *R. equi* and since the major goal of this paper is to implicate specific innate immune sensors in *R. equi* infection, we feel justified with this model moving forward. We are careful to add additional validation experiments using BMDMs throughout the manuscript whenever possible to support our conclusions (Figs. 1A, 3A, 3H)

2/ Fig 2. Agree that there is a higher induction of these genes by 8 h (the latest time point measured) but it cannot be concluded that the peak of production was in this time point as later time-points were not studied. It will be also important to report the expression levels of their control (*Actb*) to fully understand how much is being produced, as well as report the data with the same y-axis graphs. If graphs in 2F had the same y-axis, will facilitate seeing that Liff1 is expressed higher in RAW264.7 cells and in primary BMDMs, and primary BMDMs have higher ISG expression that RAW264.7 (at least at 8 h) indicating that RNA-seq data at 8 h in primary BMDMs may be a better read out for this analysis.

We have amended the language in this section to reflect that the induction of these genes is higher and removed the statement regarding *peak* production (line 215). We have also included expression levels of our control (*Actb*) in the supplemental figures (Supplemental Figs. 1B-E, 2B, 3A, 3E, 4H, 4L-M) and all gene expression studies are normalized to *Actb* (Figs. 1A-C, 1E-F, 2D, 3A-B, 3D-E, 3G-K, 4B-E, 5A-B, 5E-H, 7C-D, 8A-C). Finally, we have adjusted similar graphs so that they are on the same y-axis (Figs. 1A, 1C, 1F, 3D-E, 3G).

3/ Fig 3. It seems that although not significant (probably due to the n values reported and variability, large error bars), *Ifnb* has a higher difference in expression at 4 and 8 h than il6 does, although the last one is significant. It will be important to have all graphs with the same y-axis scale for comparative reasons. Is there are *R. equi* overexpressing VapA? It is not clear the expression levels of VapA in 33701+. Data in Fig 2D and Fig. 3B for *Infb* seems to be the same data set represented differently, if this is the case, authors could combine the results in the same graph to avoid confusions. Due to the minor differences in expression levels (although significant), to rule out that VapA is not being required for induction of Type I interferons, it may need to be necessary looking at protein and looking at both IFN-alpha and IFN-beta, as these are shown to be differentially expressed in these cells infected with the same virus.

As suggested, we have made all graphs with the same y-axis scale (Figs. 1A, 1C, 1F, 3G). *R. equi* 33701+ is considered the wild type strain for our experiments and is not overexpressing VapA. Unfortunately, we are not able to access VapA overexpression bacteria, although we agree this would be a powerful tool. We have included the expression levels of VapA in 33701+ and 33701- (Fig. 3C) as well as our housekeeping gene, 16S (Supplemental Fig. 2A). Overlapping data have been consolidated into one graph (Fig. 3D). We have also included an ISRE reporter assay as a readout for IFNα/β protein secretion in 33701+ and 33701- that supports the transcriptional data (Fig. 3F). Finally, to further rule out VapA in inducing type I IFN, we measured *Ifna4* and *Ifna7* and observed no difference in expression between macrophages infected with *R. equi* expressing VapA and those not expressing VapA (Fig. 3K).

4/ Fig S2. The presentation of this data set in the text may be misplaced and figure mislabeled. Further, completely abrogation is not observed for lsg15 in Fig. S2B, it is seeing a reduction related to scrabble treated cells. The same for Ifnb levels, these are reduced at 4 h under one condition. Revise the discussion of this figure in the text. Revise data related to Supplemental S2A to make it more comprehensible to the general reader. Explain why Trif signaling is measured as a strong inducer of type I IFNs and regulator of gene expression of RANTES, IP-10, MCP-1 and IL-12p40 in response to the upstream TLRs.

Yes, thank you, this was indeed an outdated draft of the figure and has been replaced with the correct figure. The text has been revised to make it more comprehensible and we expanded our explanation of why we tested TRIF as a potential inducer of type I IFN (lines 260-262).

5/ The cGAS/STING/TBK1 axis is already established for M. tuberculosis. Similarly, is there an interlink between the DNA (Esx-1 dependent) and RNA (SecA2/Esx-1 dependent) sensing pathways in stimulating Type I interferons as published for M. tuberculosis?

This is an excellent point, and the question of a link between the DNA and RNA sensing pathways in stimulating type I IFN is one we are interested in answering. Our findings that both cGAS and STING KO macrophages have markedly reduced or ablated type I IFN suggests that DNA sensing is the primary mechanism of type I IFN signaling during *R. equi* infetion. We agree that RNA sensing could be a factor, and **we mention this caveat in lines 456-458 in the discussion**. Because we do not specifically focus on RNA sensing in this manuscript, we feel that studies of RNA sensing fall outside the scope of this report. This is a very interesting topic to us though, and it is possible that RNA sensing is involved either at later time points during infection OR during *in vivo* infection. It is something we definitely will explore in the future.

6/ Fig 6. There is observed bacterial clumping in these experiments. In the case of M. tuberculosis, clumping drives phagocytosis and phagosome-lysosome fusion, which can leak DNA/RNA and other cell wall Ags into the cytosol. It is difficult to be certain of the colocalization of *R. equi* with galactin-3 and 8 at 4 h due to the clumping. At 8 h, showed results are more definitive. The question is what this means, is *R. equi* being killed and their DNA/RNA translocated into the cytosol, is this only happening in the galactin + phagosomes (which represent a low number of events), and is this happening after lysosomal fusion, so in the phagolysosome containing *R. equi*?

We agree, bacteria clumping makes it difficult to assess colocalization of *R. equi* with galectins. Because *R. equi*, like Mtb, are notoriously clumpy, we actually address this experimentally by adding a low speed spin step when we are prepping our bacterial cultures for infection, in order to spin out heavier clumped bacteria (a technique we use for Mtb). Your comment made us realize that we actually didn’t choose very good images to represent 4h post infection. Figure 6 now includes images that are more representative of our actual data, wherein we do not see many clumps of *R. equi* (which is more indicative of what we observe).

Your questions regarding what happens to galectin+ *R. equi* and how their nucleic acids spill into the cytosol are great ones. But frankly, this is something we still do not know about *M. tuberculosis* and we’ve been studying cytosolic DNA sensing during tuberculosis infection for over a decade. We plan to address questions related to the nature of the *R. equi*-containing vacuole in future studies. To begin to address this, we added new experiments in which we blocked bacterial *de novo* synthesis or vacuolar acidification and found that type I IFN gene expression was significantly reduced (Fig. 5G-H) suggesting that this might be an active process (and also consistent with ESX-1 secretion) (lines 325-340).

7/ Fig 7, mouse study, it is not clear why responses were not measured in the lungs, where the infection normally establishes, as well as why Type I IFNs were not directly measured. The same applies to the foal model in Fig 8.

This is an excellent point. We used an intraperitoneal infection route in our mouse model, which is associated with lower bacterial burden in the lung than a respiratory route and thus is not appropriate for cytokine assessment in the lungs. While foals were infected via intrabronchial inoculation, this was not a terminal study and thus postmortem lung tissue was not available for assessment. However, to better address the lung environment during equine infection, **we obtained equine bronchoalveolar lavage cells and measured a significant induction of *Ifnb* in these cells following infection** (Fig. 3B). While IFN-β is our type I IFN of choice for acute infection, in longer infections (over 24 hours), interferon stimulated genes (ISGs) measured in Figures 7C-D and 8A-C represent a better readout of the type I IFN response as IFN-β expression is generally reduced by this time.

8/ As the authors mentioned there are still some important questions to answer, such as if it is in reality the *R. equi’s* DNA (or RNA, or both) involved in the stimulation of Type I IFNs or it is host DNA due to induced mitochondrial damage due to the infection.

Our findings that type I IFN production is largely ablated in the absence of cGAS and STING suggest that RNA sensing is not important in the stimulation of type I IFNs during *R. equi* infection (at least at the time points tested). But as stated earlier, we agree that RNA sensing could be involved, and we mention this in lines 456-458 of the Discussion.

To your point about mtDNA, **we added a new experiment in which we used ddC to deplete mitochondrial DNA and saw no reduction in type I IFN expression**, indicating the type I IFN response is not dependent on *R. equi*-induced spillage of mitochondrial DNA into the cytoplasm (Supplemental Fig. 4I).

Reviewer #2: Two important questions remain unfortunately not addressed in this study:
Is the activation of the cGAS/STING/IFN path important for the cell autonomous immunity of macrophages or for the general fight of the immune system against *Rhodococcus equi*? Using mice or macrophages lacking cGAS or IFNAR or STING might help the authors to answer such question.

We and others (Ji, et. al., 2019) have found that C57BL/6 mice are a poor model for studying the role of type I IFN during intracellular bacterial infection as the mice produce low levels of IFN-β even when faced with a known inducer of this response. Because our IFNAR KO strain is in the BL6 background, we did not feel this experiment would shed much light on the role of type I IFN in cell autonomous control of *R. equi*. Instead, we turned to our *in vitro* models, and as you suggested, **compared *R. equi* replication in WT vs. cGAS and STING KO macrophages**. We infected control and cGAS and STING KO macrophages with *R. equi* at an MOI of 1 and measured CFUs at 1, 12 and 24 hours and observed no significant reduction in *R. equi* bacterial burden in cGAS or STING KO macrophages at any of these time points compared with our control macrophages (Supplemental Fig. 4G).

Another outstanding question from the study is if the putative ESX-encoded secretion system from *Rhodococcus equi* is able to trigger cGAS activation and phagosomal escape/destabilization? Is there a way the authors could try such approach (genetic against some putative components of this ESX?), as it would bring great scientific information regarding the general virulence components important for driving such process. However, given the current context, if no mutant or this requires a very strong tour de force, an alternative way would be to determine using various inhibitors what in phagosomal lumen helps *Rhodococcus equi* to promote membrane damages. pH is important for *F. novicida* as well M. tuberculosis-activated cGAS, is such process also involved here? Does pH, ROS modulate phagosomal destabilization and cGAS activation?

Agreed, these are all great points. At this time, an *R. equi* ESX mutant is not available; we did try extensively to get help to this end and have made a connection with Sonsi Alvarez’s lab at the University of Georgia. We are working with them to acquire the necessary plasmids and protocols to generate an *R. equi* ESX mutant. However, because *R. equi* genetics remains so challenging/not commonplace we felt that attempting such here would unnecessarily delay resubmission of the manuscript by many months and thus ruled that it was outside the scope of this manuscript. However, as we are keen to understand the mechanisms through which *R. equi* induces type I IFN expression, we performed two additional experiments that start to get at your questions. First, to test whether internalization drives *R. equi de novo* protein synthesis involved in triggering type I IFN induction, we treated *R. equi* cultures with chloramphenicol, an antibiotic capable of inhibiting *de novo* bacterial synthesis, at two different concentrations. We found that **chloramphenicol-treated *R. equi* elicited reduced type I IFN expression compared to those treated only with solvent** (Fig. 5G), indicating that *R. equi* *de novo* protein synthesis contributes to the type I IFN response in macrophages. These experiments begin to suggest that bacterial virulence factors may be required to be upregulated after bacterial internalization in order to engage the cytosolic sensors. Secondly, in order to measure how acidification of the *R. equi* vacuole impacted type I IFN induction, we treated macrophages with the vacuolar H+ ATPase inhibitor bafilomycin A1, which blocks vacuolar acidification. **Type I IFN expression was reduced in bafilomycin A1 treated macrophages compared to those treated with solvent alone** (Fig. 5H). From this we can conclude that acidification of the *R. equi* vacuole is necessary to induce a robust type I IFN response and suggests that a lower vacuolar pH is required to fully induce a bacterial virulence gene expression program. While these experiments do not directly implicate *R. equi* ESX secretion in cGAS activation and phagosomal escape, we hope they are sufficient to start to fill this “hole” in our manuscript.

**Part III – Minor Issues: Editorial and Data Presentation Modifications**
Please use this section for editorial suggestions as well as relatively minor modifications of existing data that would enhance clarity.

Reviewer #1: 9/ Line 242: This should be Fig. S3B.

Thank you for catching this, it has been corrected.


10/ There are some issues identifying figures in the text, many are wrongly identified, especially supplemental Figure S2 and Fig 6C.

Thank you for catching these mistakes. These have been corrected.

11/ Fig 6D, some pics are out of focus.

Figure 6 has been updated with higher magnification, in focus images.

12/ Figure 4: Why did not study 8 h post-infection, where the peak in all the other results was reported? How many times this was done? This is not clear in the figure legend, although this info is in the Materials and Methods section.

The majority of gene expression studies were carried out until at least 8 hours post infection. Western blot analyses were carried out until 6 hours because IRF3 is activated upstream of *Ifnb* transcription and because peak production for IRF3 was observed at 4 hours. Figure legends for Figs. 1, 3-6 have been updated to reflect the number of times experiments were repeated. Immunoblots for pSTAT1 and pIRF3 were repeated at least 3 times.

13/ The strategy of using shRNA technology in mouse cell lines to KO for example MyD88 and not use primary cells from KO mice.

Primary cells from KO mice were used for TBK1 (Fig. 4B-D) and IFNAR studies (Supplemental Fig. 3B-E).

14/ Line 253-254: Revise if 'expression' should be 'production’ and 'production' should be 'expression'.

Thank you, this has been revised as suggested.

15/ There is not need for Fig 7A and B, and Fig. 8A.

Figures 7A and 8A have been revised (removed) as suggested.

16/ Specifics about IAUC protocol numbers and foal housing is missing.

A statement regarding the approval of animal procedures by Texas A&M Animal Care and Use Committee has been included (line 493). The animal methods section has been amended to include the missing foal housing information (lines 497-499).

17/ Mouse infection study methodology is missing.

Thank you for catching this. The mouse infection study methodology has been included (lines 573-583 of methods section).

Reviewer #2: A minor point, but interesting, would be testing in primary human macrophages is *Rhodococcus equi* triggers cGAS-dependent IFN production and or cGAS-dependent macrophages necrosis as it has been shown that cGAS can mediate both through STING activation in primary human macrophages. https://www.cell.com/cell/pdf/S0092-8674(17)31133-9.pdf

In lieu of primary human macrophages, we added experiments using the human monocyte cell line U937 to show that *R. equi* triggers cGAS dependent type I IFN production in human macrophages in Fig. 5C-D.

Reference

Ji DX, Yamashiro LH, Chen KJ, et al. Type I interferon-driven susceptibility to Mycobacterium tuberculosis is mediated by IL-1Ra [published correction appears in Nat Microbiol. 2020 May;5(5):777]. *Nat Microbiol*. 2019;4(12):2128-2135. doi:10.1038/s41564-019-0578-3
